# Ocular Rosacea microBiome Study (ORBS)—sub-microbial versus antibiotic dosing of doxycycline versus placebo in treatment of symptomatic ocular rosacea: study protocol for a parallel-arm randomized clinical trial

**DOI:** 10.1186/s13063-022-06948-9

**Published:** 2022-12-20

**Authors:** Hamidah Mahmud, Jeremy D. Keenan, John Gonzales, Julie Schallhorn, Matilda Chan, Benjamin Arnold, Victoria Cavallino, Thomas M. Lietman, Thuy Doan, Gerami D. Seitzman

**Affiliations:** 1 San Francisco School of Medicine, University of California, San Francisco, CA USA; 2 Francis I. Proctor Foundation, University of California, San Francisco, CA USA; 3 Department of Ophthalmology, University of California, San Francisco, CA USA; 4 Department of Epidemiology and Biostatistics, University of California, San Francisco, CA USA

**Keywords:** Ocular rosacea, Microbiome, Doxycycline, Sub-microbial, Antimicrobial resistance

## Abstract

**Background:**

Ocular rosacea is common and is often managed with long-term antibiotic treatment. Doxycycline is the most commonly selected antibiotic for the treatment of rosacea. As there is no established standard of care treatment dose for rosacea, prescribed doses of doxycycline vary widely. The FDA classifies 40 mg daily dose of doxycycline for ocular rosacea as sub-microbial in comparison to an antibiotic dose of 200 mg daily. However, this “sub-microbial” dose has never been evaluated in patients with ocular rosacea, and even the sub-microbial dose has potential to alter systemic mucosa flora. Here, we present a randomized controlled trial using RNA sequencing to fully characterize the impact of sub-microbial antibiotic dosing of doxycycline on antimicrobial resistance and bacterial composition of the ocular and gut flora.

**Methods:**

In a triple-masked parallel randomized control trial, patients with ocular rosacea will be randomized to three arms: a 40-mg dose of doxycycline, a 200-mg antibiotic dose of doxycycline, or placebo. Collected rectal and lower eyelid samples will be compared for frequency of antimicrobial resistance genetic determinants and microbiome diversity. A subjective ocular surface disease index survey and objective tear breakup time measurement will be determined.

**Discussion:**

These results will enhance our understanding of the overall systemic impact of long-term systemic sub-microbial antibiotic dosing for the treatment of chronic recurrent ocular inflammatory diseases.

**Trial registration:**

This trial was registered on ClinicalTrials.org (NCT05296837) on March 22, 2022.

**Supplementary Information:**

The online version contains supplementary material available at 10.1186/s13063-022-06948-9.

## Introduction

**Fig. 1:**
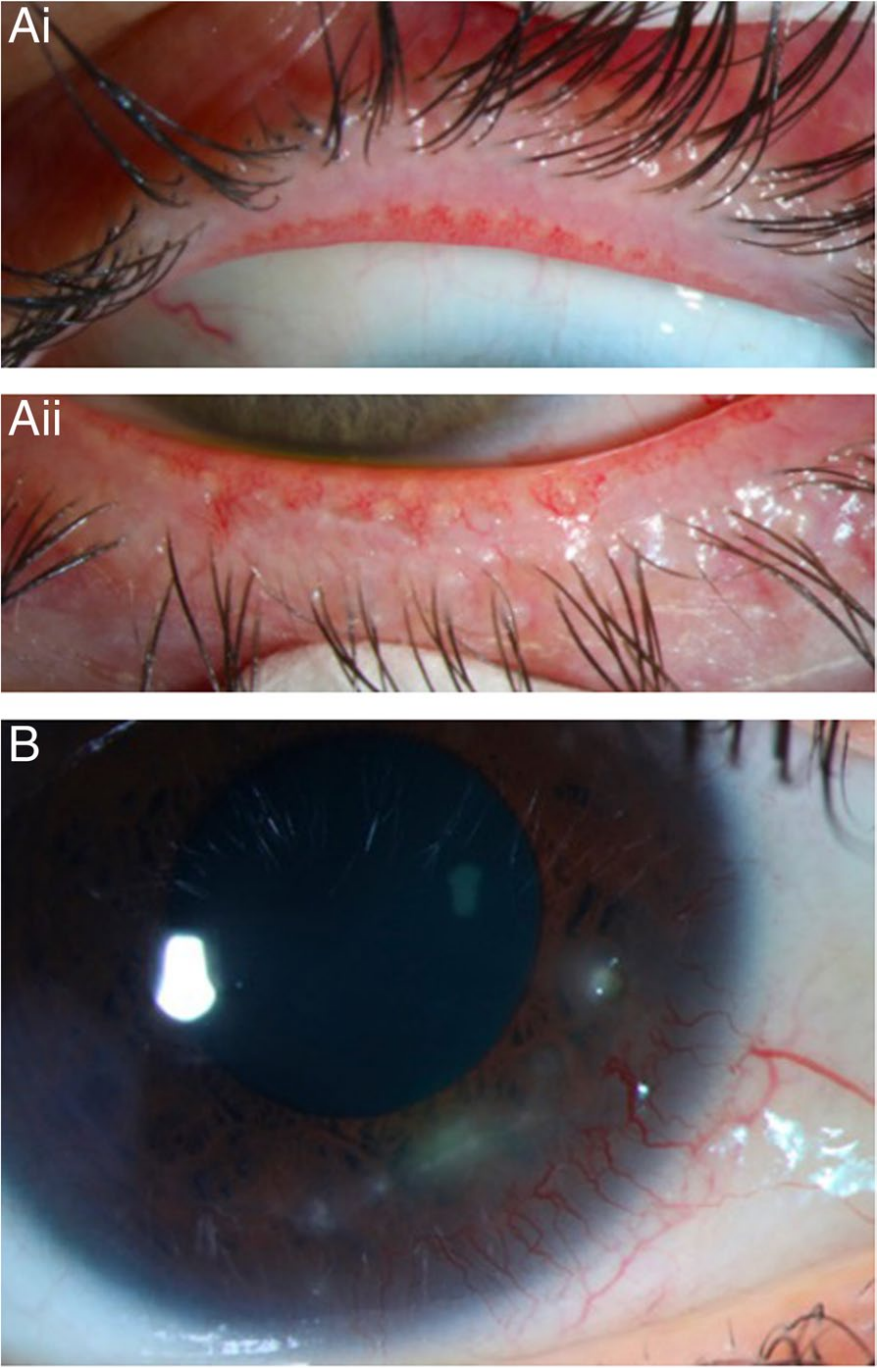
Clinical examples of ocular rosacea. **a** Eyelid margin findings in a patient with ocular rosacea: upper (i) and lower (ii) eyelids with lid margin telangiectasia and Meibomian gland clogging. **b** Corneal neovascularization and peripheral keratitis in a patient with ocular rosacea

Ocular rosacea is an inflammatory disease of the eyelids and ocular surface [1]. Like the facial disease, the ocular condition is chronic and recurrent. Sequelae of ocular rosacea vary from mild to severe. Ocular rosacea may cause chronic eye redness, blepharitis, recurrent chalazion dry eye, corneal erosion, corneal vascularization, and corneal ulceration (Fig. 1). Resulting corneal opacity can result in vision loss [2].

Topical antibiotic and anti-inflammatory eye drops and ointments can be used to control mild ocular rosacea. However, severe disease, or rosacea that is not well controlled with local treatments, is treated systemically [3]. The most commonly used systemic treatment for rosacea is the bacteriostatic antibiotic doxycycline [3, 4]. Rosacea treatment regimens of doxycycline vary widely [5]. Treatment-dose doxycycline for systemic infections is 200 mg a day. However, as rosacea is considered an inflammatory disease, doxycycline is often dosed at what is termed, sub-microbial dose doxycycline (SDD). Initially introduced in the oral medicine literature, SDD are doses 40 mg and lower because systemic administration at this dose does not appear to alter the oral mucosa flora or increase resistance rates when given long-term for periodontal disease [6, 7]. Whereas 200 mg doxycycline, even when given short term, may increase the percentage of culturable nasopharyngeal flora that is resistant to doxycyline [7, 8]. The FDA does not categorize SDD an antibiotic, stating this dosing is expected to exhibit only anti-inflammatory activity [6].

SDD is widely used for the treatment of rosacea, and though previous studies have shown a 40 mg dose does not affect oral flora microbiome and resistance, further research on other systemic mucosa’s flora is needed [6]. Data provided by the manufacturer showed that administration of 20 mg doxycycline twice daily to healthy adults resulted in concentrations of antimicrobial-resistant bacteria below the minimum antimicrobial level at 24 h after administration [8].The classification of 40 mg as “sub-microbial” has never been evaluated in patients diagnosed with ocular rosacea. Additionally, confirmation of a “sub-microbial” dose has not been investigated with more sophisticated recently available genomics and resistance tools such as unbiased RNA deep sequencing (RNA-seq).

The goal of this proposal is to use RNA-seq to determine whether SDD given to patients with ocular rosacea can be truly considered sub-microbial, or if a 40 mg dose of doxycycline can in fact alter the microbiome of the ocular surface and gut and increase resistance rates when prescribed for up to 8 weeks. We plan to evaluate the effect of SDD on antimicrobial resistance and microbiome alteration through a randomized controlled masked trial.

The objectives of this trial are to investigate if there are detectable alterations in tetracycline antimicrobial resistance gene expression and microbiome of the ocular surface and gut with administration of SDD for the treatment of ocular rosacea. Our specific aims are to investigate (1) alterations of antimicrobial resistance (AMR) in the gut and ocular surface, (2) alterations to the gut and ocular surface microbiota, and (3) changes in conjunctiva immune response expression. We hypothesize equivalency in that doxycycline at both the antibiotic treatment dose (200 mg) and SDD dose (40 mg) will increase tetracycline resistance gene expression, alter microbiota, and alter immune response genes relative to placebo.

## Methods

### Study design

The Ocular Rosacea microBiome Study (ORBS) is a three-armed, parallel, randomized, masked, placebo-controlled clinical trial. Participants with a diagnosis of symptomatic ocular rosacea (*n* = 50) at the University of California, San Francisco in San Francisco, California, United States, will be prospectively enrolled and randomized to one of three arms in a 2:2:1 fashion. (1) Arm A, a SDD dose of doxycycline (40 mg) taken for 8 weeks; (2) Arm B, an antibiotic dose of doxycycline (200 mg) taken for 8 weeks; and (3) Arm C, a placebo pill taken for 8 weeks. The trial will begin enrollment in December 2022. This protocol adheres to the Standard Protocol Items: Recommendations for Interventional Trials (SPIRIT) guidelines (Supplementary Table S1) and is registered on clinicaltrials.org (NCT05296837) [9]. This study was reviewed and approved by the Institutional Review Board at the University of California, San Francisco (21–34725) and will be done in accordance with the Declaration of Helsinki.

### Setting, participants, and eligibility

The study setting for this trial is the ophthalmology clinics at the F.I. Proctor Foundation and Department of Ophthalmology and the dermatology clinics at the University of California, San Francisco (UCSF). Participant recruitment will include treating clinicians referring patients with ocular rosacea to one of the study staff listed on the protocol. The study coordinator will review the patient’s clinical information to determine if they are eligible for the study. The study staff members will approach, and consent interested participants for the study.

Inclusion criteria for this study include if the participant has (1) symptomatic ocular disease attributed to ocular rosacea as the primary diagnosis, (2) the ability the give informed consent, and (3) is age 18 years old or older as doxycycline is contraindicated in children under 10 years old. Patients with ocular rosacea will be excluded from study participation if they have (1) an active ocular or systemic infection, (2) a known allergy or intolerance to tetracycline antibiotics, (3) prior use of oral antibiotics within the last three months, or (4) pregnancy or the possibility of becoming pregnant within the 8-week study medication timeline as tetracyclines are considered teratogenic.

### Intervention and sample size

The study coordinator will offer a sealed container to each participant during their primary visit. Each day’s dose will be marked in individual AM/PM packets. The coordinator will instruct the patients to take both doses, AM and PM, daily for 8 weeks. Participants taking 40 mg doxycycline will be taking one 20 mg doxycycline pill twice a day. Participants taking 200 mg doxycycline will be taking 100 mg twice a day. Participants allocated to placebo will be taking a placebo pill twice daily. The initial dose will not be offered under clinical observation as the pills for each study arm will look different and can unmask the participant to the study team. Participants will bring medication to all follow-up visits, compliance will be monitored by pill count performed by the study monitor. Monitoring for systemic side effects will be done at study follow-up visits via a questionnaire. We will not take objective measurements of adherence such as blood levels due to inefficiency and cost in this modest sample cohort.

We estimate that twenty patients per arm will provide over 80% power to detect a twofold increase in tetracycline resistance genetic determinants between Arms A and B, assuming the standard deviation in read number found previously, allowing for 10% dropout, and correcting for baseline [10]. As a secondary analysis, we estimate that twenty patients in Arm A and ten in Arm C will provide over 80% power to detect a three-fold increase in tetracycline resistance genetic determinants.

We estimate that twenty patients per arm will provide over 80% power to detect a difference between Arms A and B in Shannon’s diversity effective number of 2 (from an effective number of 10. That is, 10 to 8 or 10 to 12 effective number), assuming 10% drop-out and a standard deviation in the effective number of 1.9, found in two previous studies [11, 12]. As a sensitivity analysis, we anticipate similar power using Simpson’s diversity (again, expressed as an effective number).

### Randomization and masking

The study statistician will create a permuted block allocation sequence in R Foundation for Statistical Computing, to prevent overt imbalance in this modest-sized trial. The clinical study coordinator will then perform study randomization according to the 2:2:1 randomization schedule using the REDCap (Research Electronic Data capture) tool randomization module to allocate patients to groups. All study arm medications will be centrally located in sealed containers that the study coordinator will hand out according to randomized allocation. We will use letters, A, B, and C, to mask treatment arms. All patients will be masked to their SDD allocation and informed that they will be taking a pill by mouth twice a day. All examining physicians responsible for tear breakup time (TBUT) determination and ocular photography will be masked to patient allocation assignment. One biostatistician will generate the randomization key and another will perform outcome analysis masked to patient allocation assignment. Only the study coordinator, one biostatistician, and one study coordinator from a separate project will have access to the randomization key. Emergency unblinding is acceptable if a participant experiences an adverse event.

Doxycycline at 40 mg and 200 mg and placebo will be purchased from the vendor, Amerisource Bergen.

### Outcomes assessment, schedule, and procedures

The primary outcome of interest is comparing the load, a quantitative measure, of AMR genetic determinants in lower lid and rectal swab samples between arms A (SDD dose) and arm B (placebo) when corrected for baseline. The secondary outcome of interest is microbiome diversity of the ocular surface and gut through Shannon’s Index and Simpson’s index. Other exploratory outcomes include comparative analysis of (1) host conjunctiva inflammasome expression; (2) Ocular Surface Disease Index (OSDI), with scores ranging from 0 to 100 with higher scores indicating greater symptom severity; and (3) TBUT scores with numbers representing how many seconds after a full blink a discontinuity in the tear film appears. A normal tear breakup time is greater than 10 s of tear stability between blinks. All outcomes will be compared between the three study arms, corrected for baseline. Using R statistics tool, we will use ANOVA and chi-squared to measure aggregate quantitative and distributive qualitative values respectively in each arm and compare at the 4-week, 8-week, and 4-month mark. Both primary and secondary outcome analysis will emphasize differences noted at 4 months between study arms when corrected for baseline.

Participants will be seen for four approximately 40-min visits over the course of the study: at baseline, 4 weeks, 8 weeks, and once 4 months after their last dose of study medication. The four-week visit will be 25–35 days after the initial visit, the 8-week visit 49–63 days after the initial visit, and four months 90–150 days after the initial visit. Participants will undergo ocular evaluation at every visit as well as have a lower lid margin swabs from each eye and a rectal swab collected at baseline, 8 weeks, and 3 to 6 months after the first dose of study medication. Table 1 summarizes the procedure schedule for each visit under SPIRIT guidelines [9].

**Table 1:**
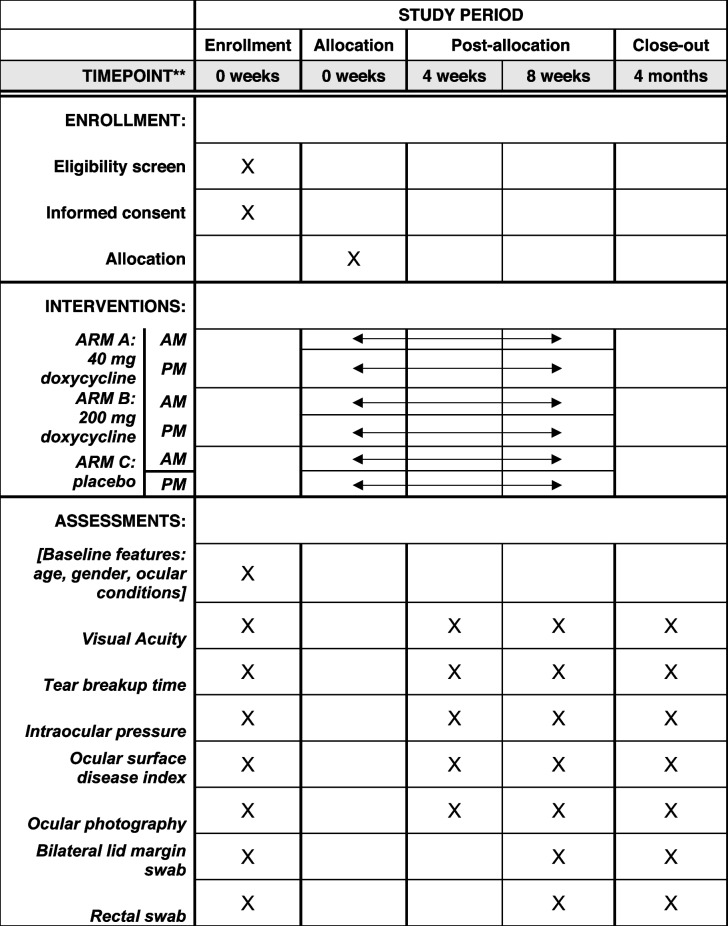
SPIRIT schedule of planned ORBS trial procedures

The following ocular evaluation procedures should be performed in the order listed below to minimize disturbance of the ocular surface.

1. Tear breakup time (TBUT)

It is preferable to get an automatic TBUT measurement, such as can be obtained through an Oculus keratography topographer. If this topographer is not available, then the TBUT should be measured by a physician masked to the study assignment. TBUT should be measured prior to application of anesthetic.

If in the case a doctor is measuring the TBUT, they should utilize the following procedure:

I. A fluorescein strip, only gently moistened, should be touched lightly to the tear meniscus.
II. The patient instructed to blink several times.
III. After even distribution of the fluorescein, the patient should be asked to blink again and then hold the eye open and the doctor starts a stopwatch.
IV. The tear film is examined under cobalt blue light at the slit lamp.
V. The doctor stops the stopwatch when the first break in the tear film occurs.
VI. This measurement is repeated a total of three times, and all three times recorded.

2) Intraocular pressure (IOP)

Non-contact tomography, such as iCare, is preferred as it requires no anesthetic that will alter the tear film. If iCare is not available, then traditional Goldmann applanation tonometry, as is routine with all eye examinations, may be performed at the end of the examination.

3) Ocular Surface Disease Index (OSDI)

The patient will complete the standard 12-question questionnaire created by the Outcomes Research Group at Allergan Inc. (Supplementary S2).

4) Ocular photography

Lid margin and conjunctiva will be photographed either with a slit lamp camera or with the ocular keratography topographer that has lid imaging modalities.

Sample collection procedures for the bilateral lower lid and rectum are summarized below. Study coordinators will provide separate sample collection kits for the lower lid and rectal samples. Samples will be processed in the Ralph & Sophie Heintz Laboratory at the Proctor Foundation for RNA deep sequencing with subsequent bioinformatic analysis.

1. Bilateral lower lid swab collection

The study coordinator will obtain patient consent before collection. A sample collection tube labeled with the patient’s ID sticker will be filled with 1 ml of Zymo solution to stabilize the RNA/DNA in the sample. The collection kit will also contain an applicator swab, tube rack, and parafilm to prevent leakage after collection.

**Fig. 2:**
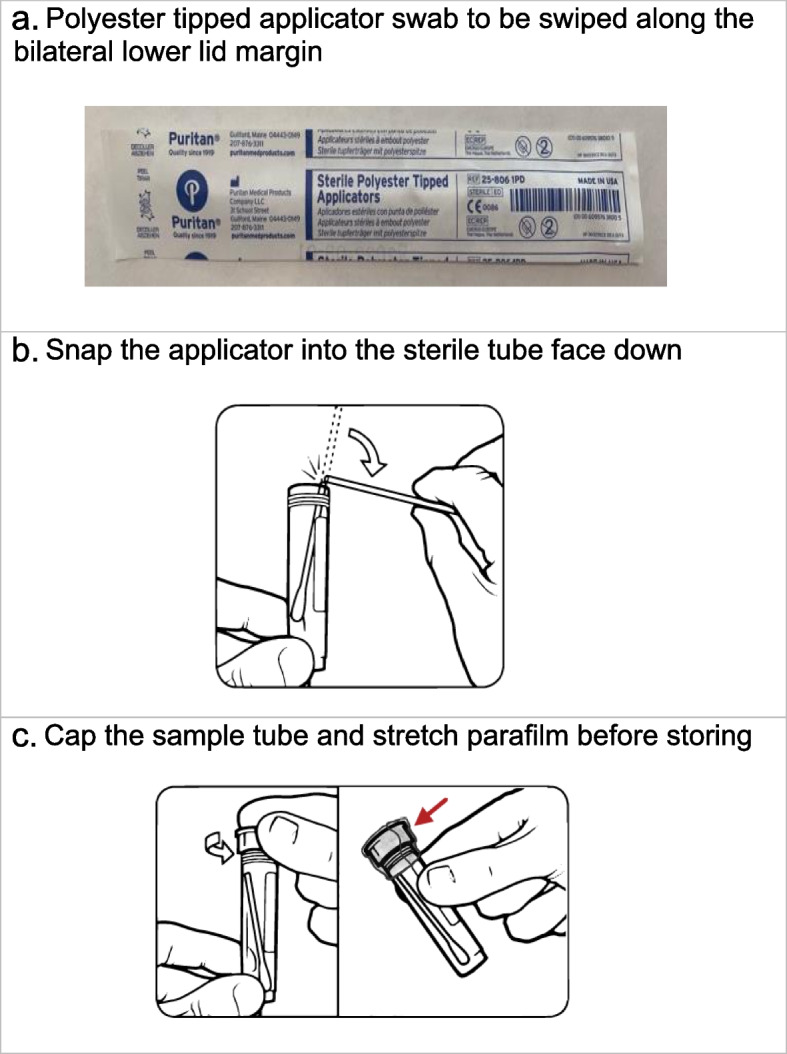
Steps in obtaining lower lid margin sample. **a** Polyester tipped applicator swab to be swiped along the bilateral lower lid margin. **b** Snap the applicator into the sterile tube face down. **c** Cap the sample tube and stretch parafilm before storing

For sample collection, the physician will wear gloves and a mask, and instruct the patient that neither of them should speak during swabbing as RNA-seq is very sensitive and can pick up oral flora contamination in the air. No anesthetic drops will be used to further limit contamination. The physician will swab the first eye using the polyester tipped applicator swab (Fig. 2a), pulling down the lower eyelid and rolling the swab across the length of the inferior lid margin twice, while rotating the swab, and then repeating in the second eye. The order of eyes swabbed is inconsequential as both eyes will be swabbed with the same applicator. The physician will then open the sterile capped plastic tube container, place the swab tip down, and carefully snap the swab while holding the tube steady with the other (Fig. 2b). Immediately after capping the plastic tube, they will stretch the parafilm over the top for further leak protection (Fig. 2c) before placing the tube back on the rack.

2) Rectal swab collection

**Fig. 3:**
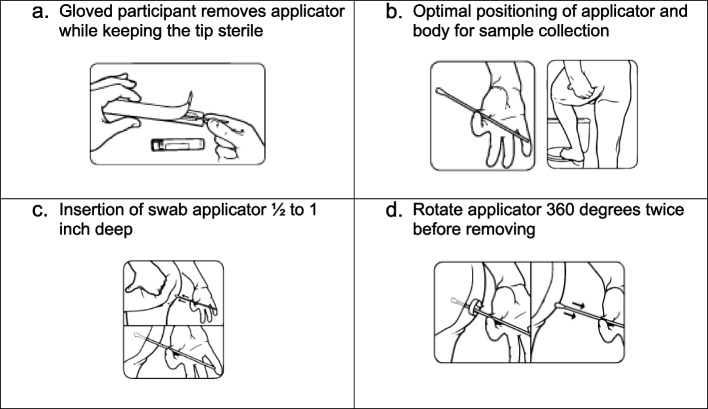
Steps in obtaining rectal sample. **a** Gloved participant removes applicator while keeping the tip sterile. **b** Optimal positioning of applicator and body for sample collection. **c** Insertion of swab applicator ½ to 1 inch deep. **d** Rotate applicator 360° twice before removing

Study participants will collect their own rectal swabs. They will first collect the swab package with a cotton-tip swab and collection tube labeled “RECTAL,” as well as a biohazard bag to place the collection swab in, from the study coordinator. In a private location such as a restroom, the gloved participant will open the sterile flocked collection device and remove the swab from its wrapper without touching the cotton tip (Fig. 3a). Holding the swab between the thumb and forefinger for the best control, they can raise one foot onto the ledge of the toilet bowl and pull back the buttock with their left hand (Fig. 3b). They will then insert the cotton tip into the anus ½ to 1 inch deep (Fig. 3c) and rotate 360° twice (Fig. 3d) before gently removing. They will then place the soft tip into collection tube, snapping the neck of the swab, firmly closing the lid, and covering with parafilm (Fig. 2b, c). After placing the tube into an empty cell of the UNLINE 6 absorbent sheet they should dispose of the gloves and thoroughly wash their hands.

### Adverse events

All adverse events will be recorded and reported via the Adverse Event Reporting Form. Serious adverse event (SAE) collection begins after the patient has signed informed consent and has taken the first dose of the study medication. Study personnel must alert the medical monitor of any SAE within 24 h of investigator awareness of the event. Alerts issued via telephone are to be immediately followed with official notification on the Serious Adverse Event Reporting form. All serious or unexpected Adverse Events judged to be definitely, possibly, or probably related to study participation will be reported to the UCSF Committee on Human Research within 5 working days of their occurrence. Causality of the adverse event will be determined by the study investigators and the Data safety monitoring committee. Participants will be compensated if harmed.

### Data collection, management, and monitoring

Demographic data will be collected on enrolled study participants including ocular conditions, age, and gender. Collected sample material will be de-identified and analyzed in aggregate. Collected data will be recorded and managed electronically using REDCap electronic data capture tools hosted at the University of California, San Francisco. All devices used for electronic data collection will be password-protected, as will the mobile application itself. To assure data quality, we will follow NIH and UCSF guidance on data management. We aim to have internal validity checks, scripts for independently reproducible data processing workflow, and document datasets in codebooks. There will be range checks for all data points and data monitoring reports to flag outliers for possible data queries. Missing or incomplete data will be noted in the data collection tool and we will utilize what is possible for an intention-to-treat analysis. We will provide the sample size (*n*) for each data point for transparency in variation.

Study data will only be accessible by study team members and investigators during the duration of the trial to protect confidentiality. Upon completion of the trial, de-identified data will be available upon request.

### Study oversight

A Data and Safety Monitoring Committee (DMSC) created before the study begins will provide independent oversight of data quality and participant safety at annual committee meetings. The committee will review the number of patients and any significant adverse events. If necessary, the study analyst will provide unmasked treatment assignment to the committee. The DSMC will recommend modifications to the study protocol as necessary. Major protocol changes will be reported to the DSMC and Institutional Review Board (IRB) as required. Important protocol amendments will be communicated to all parties including the study team and trial participants via secure email.

### Statistical considerations

The primary analysis for AMR outcomes will be ANOVA to compare determinants of resistance to doxycycline across the three study arms. Power estimates for a difference in the overall microbiome composition between Arms A and B (L1-norm PERMANOVA) are more difficult, but it is worth noting that a similar design found significant differences in the gut microbiome after oral azithromycin [11]. Individual organisms will be compared between arms using a Benjamin-Hochberg False Discovery Rate of 5%, with results considered exploratory. Both overall composition and individual species will be compared using stool and lower lid specimens. In addition, comparison of the host immune response in the lower lid between arms will be exploratory, as we do not have previous estimates of the necessary standard deviation. Although, note, this study will allow an estimate of that standard deviation for future studies.

All primary analyses will be intention-to-treat. Patients that do not attend their scheduled follow-up visits will be contacted by the study coordinator the following day via phone and offered rescheduling. Repeated attempts will be made weekly for 4 weeks to contact patients lost to follow-up. Patients who discontinue one of the study treatments due to intolerability or side effects will be observed until the end of the study period if willing to return.

An interim analysis for efficacy is not planned for this trial given a small sample size.

## Discussion

In most clinical scenarios, long-term antibiotic dosing is not recommended due to the risk of antimicrobial resistance selection [13–17]. For pediatric patients with recurrent urinary tract infections (UTIs), long-term antibiotic prophylaxis increases the risk of UTIs with antibiotic-resistant strains without significantly reducing renal scarring [18]. Long-term, low-dose macrolide treatment for pediatric patients with chronic airway diseases such as severe asthma and cystic fibrosis (CF) may have clinically beneficial outcomes, but at the cost of widespread macrolide resistance [19]. With adults, 90% of *E. coli* urinary isolates were reported resistant to trimethoprim- sulfamethoxazole within 1 month of prophylactic use and azithromycin resistance appears to be significant when used continuously to prevent acute exacerbations of chronic obstructive pulmonary disease [15, 20]. Curiously, long-term low dosing of doxycycline for ocular rosacea appears to be the universally accepted practice as this strategy is believed to bypass antimicrobial resistance, but investigation into conformation of this fact with more sophisticated genomic tools is lacking [2–4].

We note that antibiotic susceptibility determined by culture-based techniques may not fully represent the resistance profile [21]. Growing phenotypically resistant cultures in petri dishes only reveals the bacterial profile grown in culture, not the complete colonization and/ or infection of a patients’ sampled system [22]. A study using advanced genomic sequencing methods found that the presence of antibiotic multidrug resistance within the CF airway microbiome was associated with decreased microbial diversity as measured by Simpson’s index [21]. Even in cohorts of healthy patients, virome sequencing showed an expansion of antibiotic-specific resistance genes in the gut microbiome persisting three months after a 1-week course of antibiotics [23, 24]. Investigation using advanced genomic sequencing to confirm whether or not expression of AMR variants are altered in other microbiota, namely the ocular surface and the gut, has the potential to alter ocular rosacea treatment recommendations.

These results will enhance our understanding of the overall systemic impact of long-term systemic sub-microbial antibiotic dosing for the treatment of chronic recurrent ocular inflammatory diseases.

## Trial status

This study was registered on ClinicalTrials.gov on March 25, 2022 (NCT05296837). The current protocol is version 3 on April 27, 2022. There is no associated registered data set. Recruitment is estimated to begin in December 2022. The trial is estimated to be completed by December 30, 2023, and the study completed by June 31, 2024.

## Supplementary Information

**Additional file 1: Supplementary file 1.**

## Data Availability

Trial results will be presented at local, national, and international meetings and submitted to peer-reviewed journals for publication. Project involvement will determine authorship, no professional writers are anticipated to be hired. De-identified trial data will be made publicly available after publication of primary outcomes.

## Abbreviations

AE: Adverse Event
AMR: Antimicrobial Resistance
CF: Cystic Fibrosis
DSMC: Data Safety and Monitoring Committee
IOP: Intraocular Pressure
OSDI: Ocular Surface Disease Index
RNA-seq: RNA Deep Sequencing
SAE: Serious Adverse Event
SDD: Sub-microbial Dose Doxycycline
TBUT: Tear Breakup Time
UCSF: University of California, San Francisco
UTI: Urinary Tract Infection
